# Amplicon-Based, Next-Generation Sequencing Approaches to Characterize Single Nucleotide Polymorphisms of *Orthohantavirus* Species

**DOI:** 10.3389/fcimb.2020.565591

**Published:** 2020-10-14

**Authors:** Mariah K. Taylor, Evan P. Williams, Thidathip Wongsurawat, Piroon Jenjaroenpun, Intawat Nookaew, Colleen B. Jonsson

**Affiliations:** ^1^Department of Microbiology, Immunology and Biochemistry, The University of Tennessee Health Science Center, Memphis, TN, United States; ^2^Department of Biomedical Informatics, College of Medicine, University of Arkansas for Medical Sciences, Little Rock, AR, United States

**Keywords:** hantavirus, *Orthohantavirus*, NGS, next generation sequencing, MinION, illumina MiSeq, single nucleotide polymorphism, SNP

## Abstract

Whole-genome sequencing (WGS) of viruses from patient or environmental samples can provide tremendous insight into the epidemiology, drug resistance or evolution of a virus. However, we face two common hurdles in obtaining robust sequence information; the low copy number of viral genomes in specimens and the error introduced by WGS techniques. To optimize detection and minimize error in WGS of hantaviruses, we tested four amplification approaches and different amplicon pooling methods for library preparation and examined these preparations using two sequencing platforms, Illumina MiSeq and Oxford Nanopore Technologies MinION. First, we tested and optimized primers used for whole segment PCR or one kilobase amplicon amplification for even coverage using RNA isolated from the supernatant of virus-infected cells. Once optimized we assessed two sources of total RNA, virus-infected cells and supernatant from the virus-infected cells, with four variations of primer pooling for amplicons, and six different amplification approaches. We show that 99–100% genome coverage was obtained using a one-step RT-PCR reaction with one forward and reverse primer. Using a two-step RT-PCR with three distinct tiling approaches for the three genomic segments (vRNAs), we optimized primer pooling approaches for PCR amplification to achieve a greater number of aligned reads, average depth of genome, and genome coverage. The single nucleotide polymorphisms identified from MiSeq and MinION sequencing suggested intrinsic mutation frequencies of ~10^−5^-10^−7^ per genome and 10^−4^-10^−5^ per genome, respectively. We noted no difference in the coverage or accuracy when comparing WGS results with amplicons amplified from RNA extracted from infected cells or supernatant of these infected cells. Our results show that high-throughput diagnostics requiring the identification of hantavirus species or strains can be performed using MiSeq or MinION using a one-step approach. However, the two-step MiSeq approach outperformed the MinION in coverage depth and accuracy, and hence would be superior for assessment of genomes for epidemiology or evolutionary questions using the methods developed herein.

## Introduction

Hantaviruses, genus *Orthohantavirus*, family *Hantaviridae*, order *Bunyavirales*, are broadly distributed in nature in Old and New World rodents (Peters and Khan, 2002; Jonsson et al., 2010; Vaheri et al., 2013). Spillover of several of these viruses from rodents to humans through inhalation of aerosolized excreta can cause serious illness resulting in hantavirus pulmonary syndrome (HPS) in the Americas or hemorrhagic fever with renal syndrome (HFRS) in Europe and Asia with case fatality rates ranging from 1–40% (Peters and Khan, 2002). Hantaviruses have a tripartite, negative-sense single stranded RNA genome comprised of the small (S), medium (M), and large (L) segments; and are ~1,600, 3,600, and 6,500 nucleotides in length, respectively (Jonsson and Schmaljohn, 2001). Each genomic segment (vRNA) serves as the template for synthesis of positive-sense messenger RNA (mRNA) and complementary RNA (cRNA) during transcription and replication, respectively, within the cytoplasm (Jonsson and Schmaljohn, 2001). There is no evidence of polyadenylation of any of the hantaviral mRNAs. Diagnostic testing for patients suspected of having HFRS or HPS typically calls for qRT-PCR detection based on hantavirus S segment from blood, which does not typically discriminate among the viral vRNA or cRNA/mRNA (Terajima et al., 1999; Evander et al., 2007), and/or detection through IgM antibody capture (Le Duc et al., 1990; Ksiazek et al., 1995; Hujakka et al., 2003). Neither of these approaches provides information regarding the specific strain or genotype of hantavirus, whether the virus represents a new sequence variant or whether the infection represents a new reassortment of the genomes. The approaches are similar for detection of hantaviruses for ecological surveillance of small animals. However, the low copy number of hantaviral RNAs in tissues typically require a nested-RT-PCR approach which is not appropriate for whole genome sequencing (WGS) for evolutionary or epidemiological studies (Nichol et al., 1993). With these gaps in mind, we designed, tested and evaluated several WGS approaches and methods with the goal of creating a pipeline that would result in high quality sequences for the purpose of studies of ecology, evolution, or molecular epidemiology.

Over the past decade, WGS approaches have quickly advanced to enable the rapid detection of viral genotypes and drug resistant variants in patients or environmental samples. The accurate and robust assessment of the genetic structure of a virus population to confirm genetic drift or adaptation to antivirals or new hosts, and the phylogenetic relationships between virus species demands WGS pipelines with optimal coverage and minimal incorporation of PCR-based errors. Two of the most common sequencing platforms are the Illumina MiSeq, a second-generation sequencing platform capable of processing short reads (≤300 bp), and the Oxford Nanopore Technologies (ONT) MinION, a third-generation sequencer which detects ionic charges from single molecules of up to 2,272,580 nucleotides (Payne et al., 2018). The MiSeq provides low frequency variant analysis as error rates are very low and increased quality scores remove a majority of substitution errors (Schirmer et al., 2016). However, the MiSeq platform does not provide as rapid detection of viruses in specimens as the MinION, which provides a portable and real-time sequencing device that can be used for rapid diagnosis (Wongsurawat et al., 2019). However, MinION quality scores do not align to expected Phred values and as the quality score increases, the error rate is not significantly reduced (Laver et al., 2015). Unlike the MiSeq, early versions of the MinION showed excessive raw error rates reported to be as high as 40% (Goodwin et al., 2015). Although newer versions have drastically decreased error rates to where current substitution error rates are between 10 and 13% (Bowden et al., 2019). Thus, each NGS platform has limitations that must be considered for the application.

Four distinct approaches have been reported for WGS of Old World hantavirus species Hantaan virus (HTNV) and Seoul virus from patient and rodent specimens (Kim et al., 2016, 2018, 2019; Song et al., 2017). These include sequence-independent, single primer amplification (SISPA), rapid amplification of cDNA ends (RACE), target-capture using virus-specific probes, and amplicon-based approaches. Of these methods, the amplicon-based method provided the most sensitive approach in terms of detection of HTNV from wild reservoir rodent lung tissue (No et al., 2019). In 2019, No et al. reported near to complete genome coverage of HTNV using 10^2^ vRNA copies. Target capture and SISPA approaches did not show complete coverage even at 10^3^ or 10^5^ copies, respectively (No et al., 2019).

Given the reported success of the amplicon based NGS approach, we designed several WGS pipelines to identify the conditions most suitable for WGS recovery, optimal coverage depth and accuracy. We first present a one-step RT-PCR approach using the MiSeq and MinION which would be sufficient for diagnostic purposes with the intent of rapid species identification. However, within tissues, the one step RT-PCR approach would amplify all viral RNA species (i.e., vRNA, mRNA, and cRNA), which are all present in various concentrations; and because they represent distinct replication and transcript processes of the hantaviral polymerase, one cannot assume they have equivocal error rates. Hence, we designed an optimized a pipeline for WGS of the hantaviral vRNA or viral genome using a two-step RT-PCR, vRNA primer specific approach to enable robust single nucleotide polymorphism (SNP) calling. In this work, we focused our efforts solely on sequencing of the vRNAs to enable the study of viral genetic diversity. We present data with MinION and MiSeq in these studies. The WGS MiSeq pipeline developed minimized PCR bias, provided complete genome coverage, and provided high quality data.

## Materials and Methods

### Cells and Viruses

Vero E6 cells were purchased from the American Type Culture Collection. Vero E6 cells were grown in Eagle's minimal essential media (EMEM) (Corning, NY, USA) supplemented with 10% FBS (Gibco, Waltham, MA, USA), 5 mM penicillin/streptomycin (Gibco), and L-glutamine (Gibco). Cells were maintained at 37°C with 5% CO_2_.

Andes virus (ANDV) strain Chile-9717869, Dobrava-Belgrade virus (DOBV), and Prospect Hill virus (PHV) were provided by Dr. Connie Schmaljohn (USAMRIID, Frederick, MD, USA). Sin Nombre virus (SNV) strain Muerto Canyon and was provided by Dr. Christina Spiropoulou (Centers for Disease Control and Prevention). Hantaan virus (HTNV) strain Fojnica was purchased from BEI Resources (Manassas, VA, USA). PHV passage (P4), SNV (P1), DOBV (P1) and HTNV (P2) were grown in EMEM in one T175 flask of Vero E6 cells using a multiplicity of infection (MOI) of 0.1. Each flask was refed on day three post-infection and on day seven post-infection supernatant was centrifuged at 220 × *g* for 15 min to remove cellular debris. Cells were washed with DPBS and viral supernatant and cells were harvested with TRIzol LS or TRIzol reagent (Invitrogen, Waltham, MA, USA), respectively. RNA was extracted from supernatant using TRIzol LS and RNA from cell culture was isolated from TRIzol reagent following the manufacturer's protocol.

To generate a concentrated stock of ANDV (P6), 10 T175 flasks of Vero E6 cells were infected with a MOI of 0.1 for 1 h, rocking every 15 min. After 6 days, the cell monolayer was harvested in TRIzol reagent and virus supernatant was collected and virions were concentrated and purified on a sucrose cushion as described previously (Parvate et al., 2019).

### Genome Amplification

#### Amplicon Amplification Schemes

**Figure 1:**
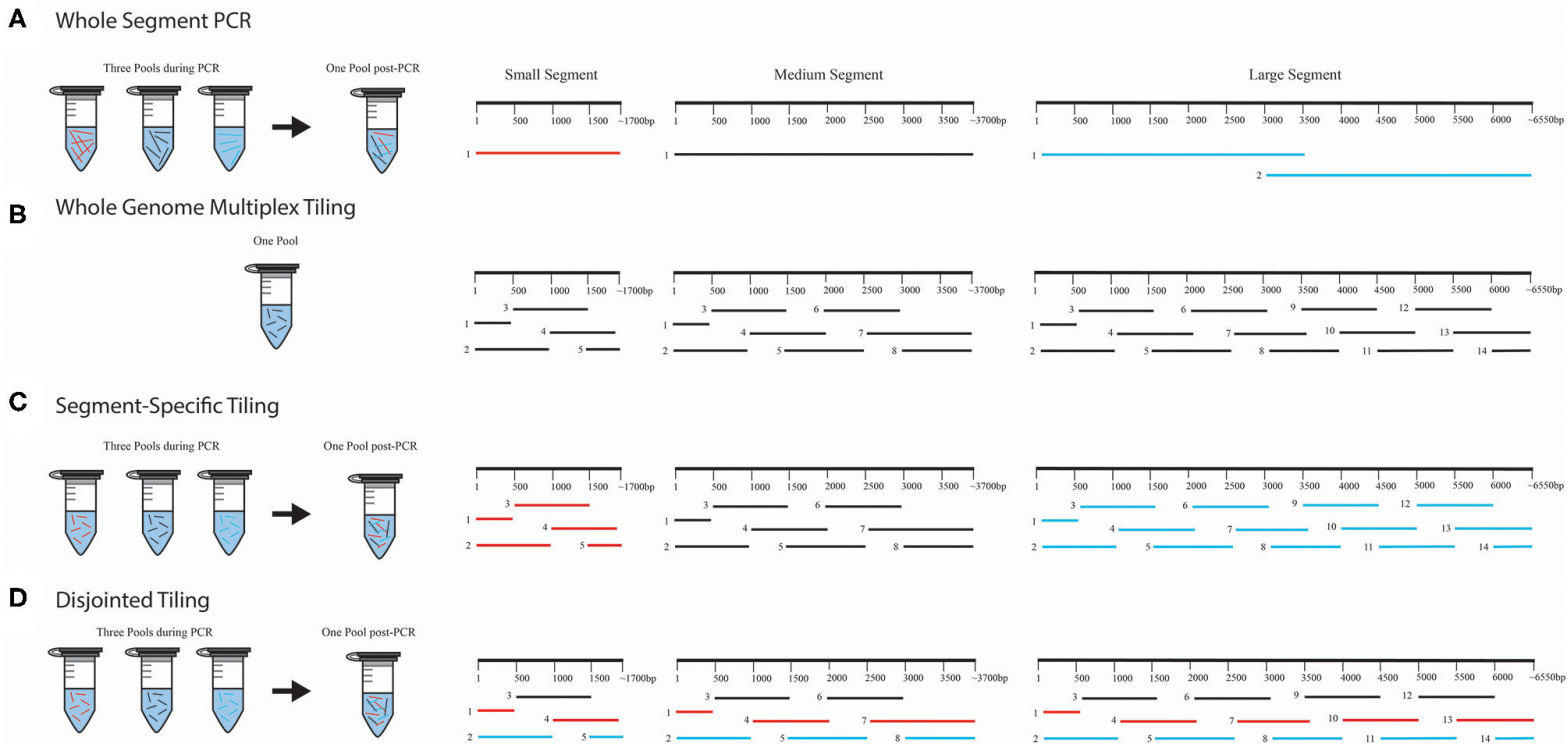
WGS amplicon-based primer amplification and pooling schema. We provide four illustrations for each method used for amplification and pooling; **(A)** whole-segment PCR approach, **(B)** whole genome multiplex tiling approach, **(C)** segment-specific tiling approach, and **(D)** disjointed tiling approach. In **(A)**, the whole-segment approach amplified the full-length genome for S and M. Two amplicons were used for ANDV L. Only the first half of DOBV and HTNV L segments were amplified with this approach. In **(B)**, each segment was amplified using overlapping primers in one multiplex reaction with all the segments in one PCR pool. In **(C)**, each segment was amplified separately and pooled for NGS library construction. In **(D)**, primers from S, M, and L were selected and multiplexed with each color (black, red, or blue) representing the PCR amplification pooling scheme. Following amplification all products were pooled for library construction.

Several approaches were used for amplification of amplicons in these experiments (Figure 1), but all primers were designed to amplify the genomic vRNAs for S, M, and L segments. For one-step RT-PCR experiments that were sequenced using the Illumina MiSeq, we used one forward and one reverse primer to amplify full-length S or M segments of ANDV, DOBV or HTNV (Figure 1A), which we refer to as a *whole segment PCR approach*. Two forward and reverse primers were used to amplify two halves of the L segment of ANDV (Figure 1A). As identification of an optimal primer set of DOBV or HTNV like ANDV was not possible at that time, we proceeded with primers that amplified only the first half of the L segment. Each amplicon was purified and combined in equimolar concentration for Nextera XT library preparation.

For the one-step RT-PCR experiments that used the MinION for sequencing we multiplexed an equimolar concentration of forward and reverse primers (Figure 1B), which we refer to as a *whole genome multiplex tiling approach*.

For the two-step RT-PCR experiments, we tiled primers across the S, M, and L genomes to amplify 1 kb segments. Each amplicon overlapped with the adjacent region by 500 bp. This approach ensures that SNPs detected within the viral population would be amplified 2X more than erroneous RT-PCR-introduced SNPs. In these experiments, vRNA-specific primers for S, M, and L segment genomes were pooled for cDNA synthesis. For the subsequent PCR amplification step, we combined an equimolar mix of forward and reverse primers (Figure 1B). We also used a *segment-specific tiling approach* (i.e., S, M, or L, Figure 1C) in which we conducted PCR reactions with pools of primers for that segment. In Figure 1C, the amplicons within each segment overlapped within other and hence each segment, S, M, or L, was amplified separately in three pools (Colored as red = S segment, black = M segment and blue = L segment).

We also tested the PCR amplification of the entire genome (included all primers for S, M, and L) in multiplexed reactions in which the primers would not generate overlapping amplicons during PCR amplification (Figure 1D). We refer to this as our *disjointed tiling approach*. Hence in each primer pool in Figure 1D represented by color as black, blue or red constituted a mixture of S, M, and L primers were multiplexed to make three amplification pools.

#### Primer Design

Primers were designed based either on GenBank consensus sequences or on the MiSeq consensus sequence of our lab reference strains and assessed using IDT's OligoAnalyzer tool, https://www.idtdna.com/calc/analyzer, under the following parameters: 0.5 μM oligonucleotide, 0 mM Na^+^, 1.5 mM Mg^++^, and 0.2 mM dNTP. Additionally, we used this tool to evaluate primer length, GC content and melting temperature. The “E value” of each primer was also assessed using NCBI Blast (https://blast.ncbi.nlm.nih.gov/) to ensure each primer was highly specific to each hantavirus species. Primer sets were tested with purified viral RNA from seed stocks by RT-PCR and run on a gel electrophoresis and only redesigned if the amplicon band did not show coverage equivocal to other amplicons or suggested the presence of primer dimers (<50 bp).

#### RNA Purification and One-Step RT-PCR Amplification for MiSeq

Total RNA was extracted from 5 ml of 10^5^ to 10^6^ PFU/ml of clarified virus supernatant from ANDV, DOBV or HTNV using 15 ml TRIzol LS per the manufacturer's instructions and 2 μL of glycogen (Ambion) per ml. RNA was resuspended in 20 μL of RNase-free water. The RNA was not quantified as the levels would be <1 pg as the weight of one genome = 6.5 × 10^−9^ ng, moreover, the RNA was not taken for real-time RT-PCR given the limited amount of material. The cDNA was synthesized from 1 μL of each RNA extract (~8 × 10^3^ PFU if 100% of the material was extracted) and amplified using SuperScript IV One-Step RT-PCR System (Invitrogen) using segment-specific vRNA primers, using the three-pool, whole segment PCR (Figure 1A) following manufacturer's instructions of 40 PCR cycles and an annealing temperature of 60°C for DOBV and HTNV or 64.6°C for ANDV. PCR products were run on a 1% agarose gel and bands were excised and purified using Wizard SV Gel and PCR Clean-Up System (Promega, Madison, WI, USA). Library preparation was performed using Nextera XT DNA Library Preparation Kit (Illumina, San Diego, CA, USA) following the manufacturer's protocol with the exception that tagmentation time was extended to 10 min and the final resuspension volume was in 15 μL resuspension buffer. Library concentrations were measured on the Qubit 4 Fluorometer (Invitrogen) and normalized to 4 nM and paired-end reads were sequenced using a MiSeq Reagent Kit v2 (300 cycles) on the MiSeq. The final MiSeq yield from sequencing the ANDV library on the 150-cycle v3 cartridge contained a total of 22.4 million paired-end reads passing filter which came from a final library concentration of 1.26 ng/μL or 3.82 nM.

#### RNA Purification and One-Step RT-PCR Amplification for MinION

RNA was purified from 50 μL of twice-clarified supernatant of PHV and SNV seed stocks (10^6^ PFU/ml) using MagMax Viral RNA Isolation kit (Applied Biosystems, Waltham, MA, USA). RNA was reverse transcribed and amplified through SuperScript IV One-Step RT-PCR System using 35 cycles. The annealing temperature for PHV and SNV primers were 60 and 66.1°C, respectively. Segment-specific primers were pooled and used in separate RT-PCRs per each viral genome segment (i.e., Figure 1C, three-pool, segment-specific tiling approach of S, M or L) or in a second method, all primers were used in one single RT-PCR (one pool, whole genome multiplex tiling, Figure 1B). Libraries were purified using Wizard SV Gel and PCR Clean-Up System. DNA library preparation was carried out using the one dimension (1D)-Native barcoding genomic DNA kit (SQK-LSK108) with the EXP-NBD103 kit (ONT, Oxford, United Kingdom) following the manufacturer's protocol with two exceptions. First, as we started from known PCR amplicon size, DNA fragmentation and DNA repair processes were ignored. Second, libraries were washed in 80% ice cold ethanol, isolated using AMPure XP beads (Beckman Coulter, Brea, CA, USA) and resuspended in 10 μL nuclease-free water. The prepared DNA library was loaded into single R9.4.1/FLO-MIN106 flow cell on a MinION Mk1B for 48 h. The final MinION yield from sequencing the PHV library provided 4.2 million reads passing filter from a final library concentration of 220 ng or 333 nM.

#### Two-Step RT-PCR and MiSeq Sequencing

The virus pellet was resuspended in TRIzol LS and RNA was extracted from supernatant as well as from cells following the manufacturer's protocols. We multiplexed vRNA-specific primers as illustrated in Figure 1 and generated cDNA using SuperScript IV First-Strand Synthesis System (Invitrogen). Forward and reverse primers were used for the second-step PCR amplification using the whole genome multiplex tiling approach (Figure 1C) or the disjointed tiling approach (Figure 1D), PCR amplification with Platinum SuperFi PCR Master Mix (Invitrogen) with 1x, 7x, 15x, and 20x amplification cycles using an annealing temperature of 64.6°C following the manufacturer's protocols.

#### Two-Step RT-PCR and MinION Sequencing

RNA was purified as stated in the prior section. The vRNA-segment-specific primer pools were used for cDNA synthesis using SuperScript IV First-Strand Synthesis System and three-pool, segment-specific amplification approach of S, M, or L (Figure 1C) or the disjointed tiling approach (Figure 1D) were used with Phusion High-Fidelity PCR Master Mix with HF Buffer (Thermo, Waltham, MA, USA) following manufacturer's protocols. PCR reactions were amplified with 7x or 30x cycles using a 60°C annealing temperature. Sample purification and library preparation was carried out as described in the one-step MinION approach as stated above.

### Bioinformatics

#### Illumina MiSeq Bioinformatics Analysis

Paired-end reads were demultiplexed on the MiSeq instrument and data was transferred to CLC Genomics Workbench v.12 (Qiagen, Hilden, Germany). Reads were quality trimmed by discarding duplicate mapped reads as well as short reads (<50 bases). The remaining trimmed reads were then aligned to previously sequenced reference genomes of each virus ANDV (accession no. SAMN14763865 of which had been aligned to GenBank reference AF291702.1, AF291703.2, AF291704.5), and GenBank references for DOBV (GU904042, GU904035, GU904029) and HTNV (X55901.1, M14627.1, M14626.1) using a length fraction of 0.9, a similarity fraction of 0.8 and keeping other settings on default. These reads were further processed by having duplicate mapped reads removed and then this final data set was used to generate a mapping report, SNPs, and generate consensus sequences. The mapping report was directly taken from the data set by excluding short contigs below a length of 200 as well as long contigs above a length of 10,000. For SNP detection, a cutoff depth of 400x and marginal variants seen below 1% were removed and a minimum quality score of 20 was used. Consensus sequences were extracted with a depth threshold of 50x or 400x. The genome coverage on consensus sequence was determined using a 50x or 400x depth of coverage threshold. Other parameters were left on default settings.

#### MinION Bioinformatics Analysis

Basecalling and demultiplexing were performed using Albacore v. 1.2.3 (ONT). The Galaxy web platform (Afgan et al., 2018) was used to trim adapters using Porechop v.0.2.3 (https://github.com/rrwick/Porechop) and fastq files were aligned to the PHV and SNV reference genomes using minimap2 (Galaxy v. 2.17 + galaxy1) (Li, 2018) where the analysis mode was set to ‘PacBio/Oxford Nanopore read to reference mapping (-Hk19)' and other parameters were set to default. PHV fastq files were aligned to a virus previously sequenced in our lab (accession no. SAMN14838478 which had been aligned to GenBank reference EF646763, X55129.1, M34011.1) and SNV was aligned to GenBank references L37902, L37903, L37904. Mapped BAM files were transferred to CLC Genomics Workbench v.20. Reads were mapped again to the reference sequence using default settings and duplicate mapped reads were removed. This data set was then used to obtain a mapping report, determine SNPs, and generate consensus sequences. To obtain percent genome coverage and average read depth a mapping report was created using a short contigs threshold of 200 and long contigs threshold of 10,000. To obtain SNPs low frequency variants were called using a required significance of 10%, a quality score of 10, and a minimum coverage depth of 50 reads, other parameters were set to default. Consensus sequences were extracted using a minimum depth of nucleotide coverage of one. Consensus sequences were altered to reflect a coding complete genome.

#### Filtering of Reads

To assess the theoretical yield of each sequencing run we used the Lander/Waterman equation which asserts that the depth of coverage is equal to the read length multiplied by the read number which is divided by genome length (Lander and Waterman, 1988). To allow for a 400x genome coverage for each of our 16 ANDV samples which have a genome length of ~12 kb, and a read length of 2 × 75, we would expect to have at minimum 32,000 reads provided per sample. To allow for a 400x genome coverage for each of our 12 PHV samples which have a genome length of ~12 kb, and a read length of ~1,000 bp, we would expect to have at minimum 4,800 reads provided per sample. As evidenced in **Tables 3**, **5**, not every sample contained this read number, and many samples had higher reads than this. Many factors affect the total output of reads allotted to each library such as short, broken reads, reads which do not contain adapters, or reads which do not pass filter are excluded from final read counts, as well as manual errors caused by uneven sample pooling.

#### Network Analyses

Consensus sequences of the coding region were aligned using MUSCLE (Edgar, 2004a,b) and these complete consensus sequence alignments were imported into PopART and run under the “Minimum Spanning Network” with epsilon value equal to 0 (Bandelt et al., 1999; Leigh and Bryant, 2015).

#### Percent Identity Matrix

MUSCLE alignments of the complete consensus sequence of each library were used to create percent identity matrices (PIM). Clustal 2.1 (https://www.ebi.ac.uk/Tools/msa/clustalo/) was used with default parameters with the exception that the “order” parameter was changed to “input” as opposed to “aligned” (Sievers et al., 2011).

## Results

### Whole Segment Primers for Amplification and MiSeq Sequencing of S and M Segments From Old and New World Hantaviruses

To develop an WGS approach on the Illumina MiSeq platform, we first assessed a one-step RT-PCR method to amplify entire hantavirus genome segments (Figure 1A). Total RNA was purified from 10^3^ PFU of ANDV, DOBV, and HTNV seed stocks. Full-length amplicons were generated for S and M segments of ANDV, DOBV, and HTNV. Two amplicons were used to cover the full-length ANDV L segment. For DOBV and HTNV only one amplicon was amplified and tested which covered the first half of the L segment (Amplicon 1 in Figure 1A, Supplementary Table 1). Amplicons were pooled for library preparation and sequenced using an Illumina MiSeq.

The length of the S segments for ANDV, DOBV, and HTNV are 1,871, 1,672, and 1,695 nucleotides, respectively. The average depth of coverage spanned from 97-1, 273x having 1,961–16,677 aligned reads which represents ~99% genome coverage (Table 1). The length of the M segments for ANDV, DOBV, and HTNV are 3,672, 3,635, 3,616 nucleotides, respectively. The average depth of coverage was from 87–977x having 3,691–30,028 aligned reads which represents ~99–100% genome coverage. Lastly, the length of the L segment for ANDV, DOBV and HTNV are 6,562, 6,532, and 6,533 nucleotides, respectively. Primers were designed to amplify amplicons that covered the genomes from nucleotides 1 to 3,216 from the 3' end of the L segment of DOBV and HTNV (Amplicon 1 of L segment, Figure 1A). Sequencing of the libraries constructed from these amplicons resulted in coverage for DOBV and HTNV of 3,388 and 5,201, respectively. The additional coverage achieved is possibly a result of the cDNA step which can result in the additional extension of the genome. The average depth of coverage was from 191–483x having 11,623–26,579 aligned reads which represents ~100% genome coverage. To summarize, the one-step RT-PCR reaction provided 99–100% genome coverage for the S, M, and L of ANDV as well as 99–100% genome coverage for S, M and partial L segments of DOBV and HTNV.

**Table 1:** MiSeq sequencing results of Nextera XT Libraries made from pooled S, M, or L segment amplicons created using Superscript IV One Step of RNA purified from seed stock supernatant for ANDV, DOBV, or HTNV.

**Virus**	**Large segment**			**Medium segment**			**Small segment**		
	**Aligned reads**	**Average depth of coverage**	**% Genome coverage**	**Aligned reads**	**Average depth of coverage**	**% Genome coverage**	**Aligned reads**	**Average depth of coverage**	**% Genome coverage**
ANDV	13,879	259x	100	3,691	87x	99	1,961	97x	99
DOBV^*^	26,579	483x	100	30,028	977x	100	16,677	1,273x	99
HTNV^*^	11,623	191x	100	19,135	559x	99	9,781	645x	99

* *Genome coverage for DOBV and HTNV L segment primers represented only the first half of the genome (1–3216 nucleotides) as the other section was not included. The data presented in this table used Figure 1A sequencing strategy. In brief, each segment was amplified with a forward and a reverse primer set. Amplicons were purified and normalized before mixing for preparation of Nextera XT libraries*.

### Full-Length Sequencing of the S, M, and L Segments of Two New World Hantaviruses Using ONT MinION

Next, we assessed the versatility of the one-step RT-PCR approach using the MinION device using a amplicon tiling schema (Figures 1B,C). Total RNA was isolated from supernatant from Vero E6 cells infected with PHV or SNV and reverse transcribed into cDNA using either a one-pool of multiplexed primers that generated tiled 500 bp overlapping amplicons across all three segments (Figure 1B, Supplementary Table 2) or a three-pool, segment-specific tiling approach (Figure 1C, Supplementary Table 2) which also resulted in 500 bp overlapping amplicons. In the segment-specific tiling approach (Figure 1C), the segment specific primer sets are colored as red = S segment, black = M segment, and blue = L segment. In this one-step reaction, amplification occurred for 35 PCR cycles. The three pools of amplicons were normalized and used to prepare libraries to be sequenced on the MinION device.

The length of the S segment vRNA for PHV and SNV are 1,673 and 2,060 nucleotides, respectively. We assessed that the one-pool, whole genome multiplexed tiling (Figure 1B) and three-pool, segment-specific tiling approaches (Figure 1C) gave an average depth of coverage ranging from 24–3,090x and a total number of reads ranging from 91–8,577 reads; representing 75–100% genome coverage (Table 2).

**Table 2:** ONT sequencing results from 1D-Native Barcoding Genomic DNA Libraries made from two different pooling approaches of S, M, or L segment amplicons created using Superscript IV One Step of RNA purified from seed stock supernatant for SNV or PHV.

**Virus**	**Pool**	**Large segment**			**Medium segment**			**Small segment**		
		**Aligned reads**	**Average depth of coverage**	**% Genome coverage**	**Aligned reads**	**Average depth of coverage**	**% Genome coverage**	**Aligned reads**	**Average depth of coverage**	**% Genome coverage**
PHV	1	92,761	7,340x	94	2,194	307x	85	1,200	379x	83
	3	4,645	356x	93	4,213	573x	100	8,577	3,090x	100
SNV	1	125	8x	50	23	5x	92	91	24x	75
	3	248	28x	69	554	153x	93	7,819	2,062x	100

*The data presented in this table used Figure 1B (1 pool) and Figure 1C (3 pools) sequencing strategy. In brief, for those samples stated as “3 pool”, the sequencing strategy (Figure 1C) used overlapping amplicons for each segment, which were purified and normalized before the 3 pools were mixed for preparation of Nextera XT libraries. In contrast, for those samples stated as “1 pool”, all amplicons were synthesized in one reaction in Figure 1B (one pool) and purified for preparation of Nextera XT libraries*.

The length of the M segments for PHV and SNV are 3,707 and 3,696 nucleotides, respectively. We assessed that the one-pool, whole genome multiplexed tiling (Figure 1B) and three-pool, segment-specific tiling approaches (Figure 1C) gave average depth of coverage ranging from 5–573x and a total number of aligned reads ranging from 23–4,213 representing 85–100% genome coverage (Table 2).

The length of the L segments for PHV and SNV are 6,559 and 6,562 nucleotides, respectively. We assessed that the one-pool, whole genome multiplexed tiling (Figure 1B), and three-pool, segment-specific tiling approach (Figure 1C) gave an average depth of coverage ranging from 356–7,340x for PHV and 8–28x for SNV and the total number of aligned reads ranged from 4,645–92,761 aligned reads for PHV and 125–248 aligned reads for SNV, representing 93–94 and 50–69% genome coverage, respectively (Table 2).

In summary, with the exception of the L segment of PHV and SNV, PHV had higher total reads, greater genome coverage and depth of coverage in every genome segment when the library preparation used the three-pool, segment-specific tiling approach (Figure 1C) as opposed to the one-pool whole genome multiplexed tiling (Figure 1B).

### Two-Step Amplification of Full-Length Sequences of S, M, and L Segment vRNA of ANDV Using MiSeq From ANDV-Infected Vero E6 Supernatant or ANDV-Infected Vero E6 Cells

While one-step RT-PCR is a quick method, it does not distinguish among viral vRNA, mRNA, or cRNA. To amplify vRNA or cRNA/mRNA requires a primer specific, two-step RT-PCR approach to synthesize the cDNA and then amplification. A second consideration in development of a robust WGS pipeline is the associated error of the process. We know that the use of nested approaches in both one-step and two-step methods can lead to error-prone polymerase-based SNP incorporation (Kugelman et al., 2017). Due to this polymerase-based error and the need for amplification, Wang et al. determined that identification of minor variants from a virus population requires a minimum depth of 400x coverage across the genome to recognize variants found at a 1% SNP frequency (Wang et al., 2007; Ladner et al., 2014). To address this fundamental problem, we generated primer sets that would create amplicons that tiled across each segment with a 500 bp overlap. For the ends an additional primer set was employed of only 500 bp. Using this approach, the genome will be synthesized twice providing a more accurate representation of the genome. Secondly, we hypothesized that the number of PCR amplifications may contribute to introduction of error. Hence, we examined 1x, 7x, 15x, or 20x PCR of the identical cDNA synthesized using primers specific for the vRNA and measured the SNPs and apparent error. We also assessed two primer pooling schemes (Figures 1C,D, Supplementary Table 2).

**Figure 2:**
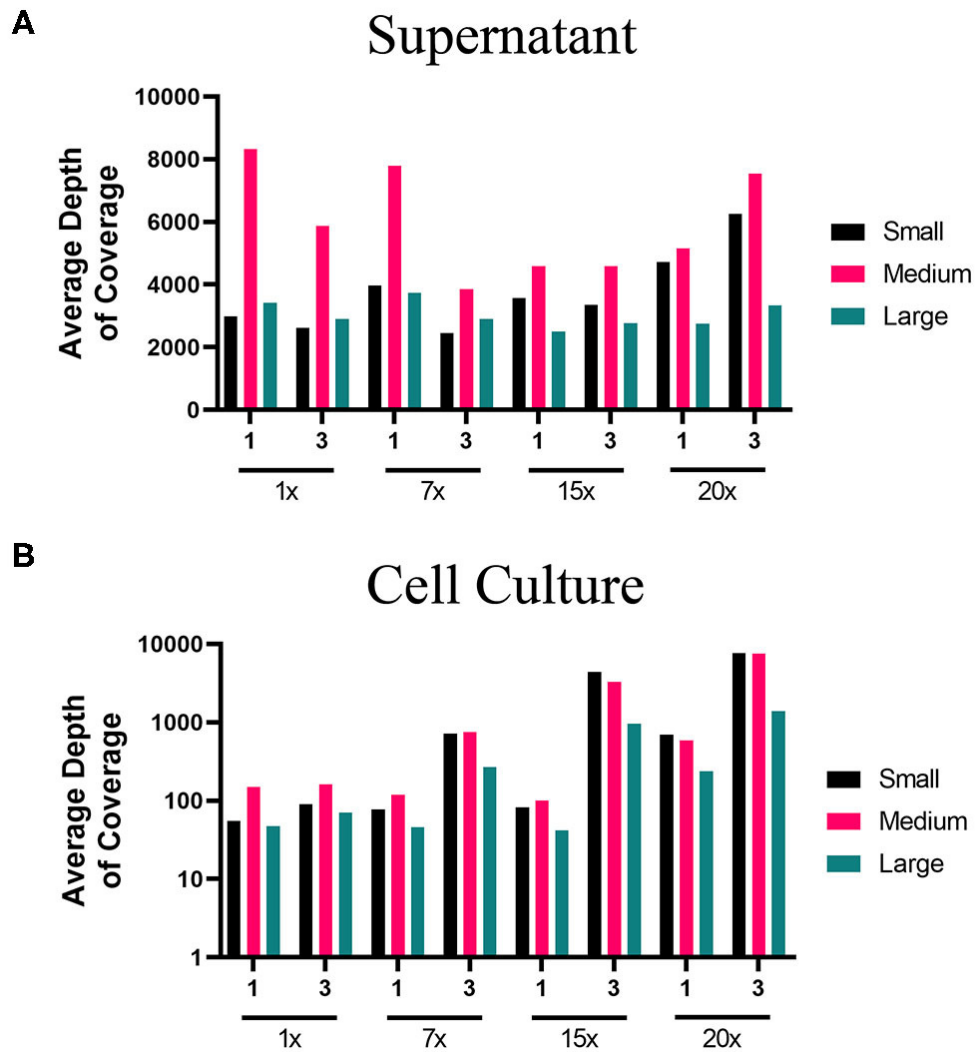
Average depth of coverage obtained from two-step MiSeq sequencing of ANDV. Illustration shows **(A)** average depth of coverage obtained for each segment (S, M, L) of ANDV RNA isolated from cell culture supernatant using different amplification approaches (1x, 7x, 15x, or 20x PCR cycles). **(B)** Average depth of coverage obtained for each segment of ANDV RNA isolated from infected cells using different amplification approaches (1x, 7x, 15x, or 20x PCR cycles).

The lower PCR amplification cycles (1x and 7x) of samples originating from supernatant provided the greatest coverage (Figure 2, Supplementary Table 3). In contrast, the higher PCR amplification cycles (15x and 20x) isolated from virus-infected cells provided greater coverage (Figure 2, Supplementary Table 3). Using a 1x depth of coverage threshold we obtained complete genome sequences from S, M, and L segments. Interestingly, this experiment resulted in near to complete genome coverage across the S and M segments. The L segment was completely covered across each primer pooling approach and each PCR cycle. Normally the L segment has a greater loss of coverage than the S or M which shows that this primer pooling approach was beneficial for complete L segment identification.

In contrast to the clarified supernatant of virus-infected cells, most diagnostic specimens are from tissue. Tissue will not only contain viral RNA, but viral mRNA and cRNA as well as host RNA molecules (rRNA, mRNA, tRNA, etc.). Host RNAs make up 1.1% of the total weight of a single mammalian cell (Alberts et al., 2008) and greatly outnumber viral RNAs. The host cell RNA can be reverse transcribed and amplified erroneously through nonspecific primer binding which increase sequencing background and result in less sequence reads aligning to viral genomes. Hence, we tested parameters to optimize viral RNA amplification from infected cells. As previously described for ANDV-infected Vero E6 supernatant, we sequenced in parallel libraries made from ANDV-infected Vero E6 cells to determine the extent by which host cellular RNA would inhibit complete viral genome recovery.

Total RNA isolated from the cell monolayer provided a slightly lowered percent genome coverage of ANDV vRNA than that isolated from supernatant. ANDV amplified from cell culture contained 99–100% genome coverage for the S, 96–100% coverage for the M, and 100% coverage for the L which is compared to 100% coverage for the S, M, and L taken from supernatant (Supplementary Table 3).

We used a 400x depth of coverage threshold to assess ANDV genome coverage (Table 3). The supernatant samples which gave the highest genome coverage across all segments was the 1x PCR and 7x PCR. The samples from infected cells showed good coverage at 15x and 20x PCR cycles although this was only true for the S and M segments as the L segment provided no coverage at this depth.

**Table 3:** Percent genome coverage of ANDV Illumina MiSeq run using a 400x minimum coverage depth.

**Sample source**	**PCR cycle**	**Pool**	**Large segment**	**Medium segment**	**Small segment**
			**% Genome coverage**		
Supernatant	1x	1	100%	96%	98%
		3	100%	97%	96%
	7x	1	100%	96%	99%
		3	0%	98%	99%
	15x	1	0%	83%	99%
		3	0%	82%	99%
	20x	1	0%	82%	99%
		3	0%	96%	98%
Cells	1x	1	0%	0%	0%
		3	0%	0%	0%
	7x	1	0%	0%	0%
		3	0%	0%	0%
	15x	1	0%	0%	0%
		3	0%	82%	97%
	20x	1	0%	0%	0%
		3	0%	82%	96%

*For those samples stated as “3 pool”, the sequencing strategy (Figure 1D) used overlapping amplicons for each segment, however primers were placed into one of three disjointed primer pools. The multiplexed amplicons were purified and normalized before the 3 pools were mixed for preparation of Nextera XT libraries. In contrast, for those samples stated as “1 pool”, all amplicons were synthesized in one reaction in Figure 1B (one pool) and purified for preparation of Nextera XT libraries*.

**Figure 3:**
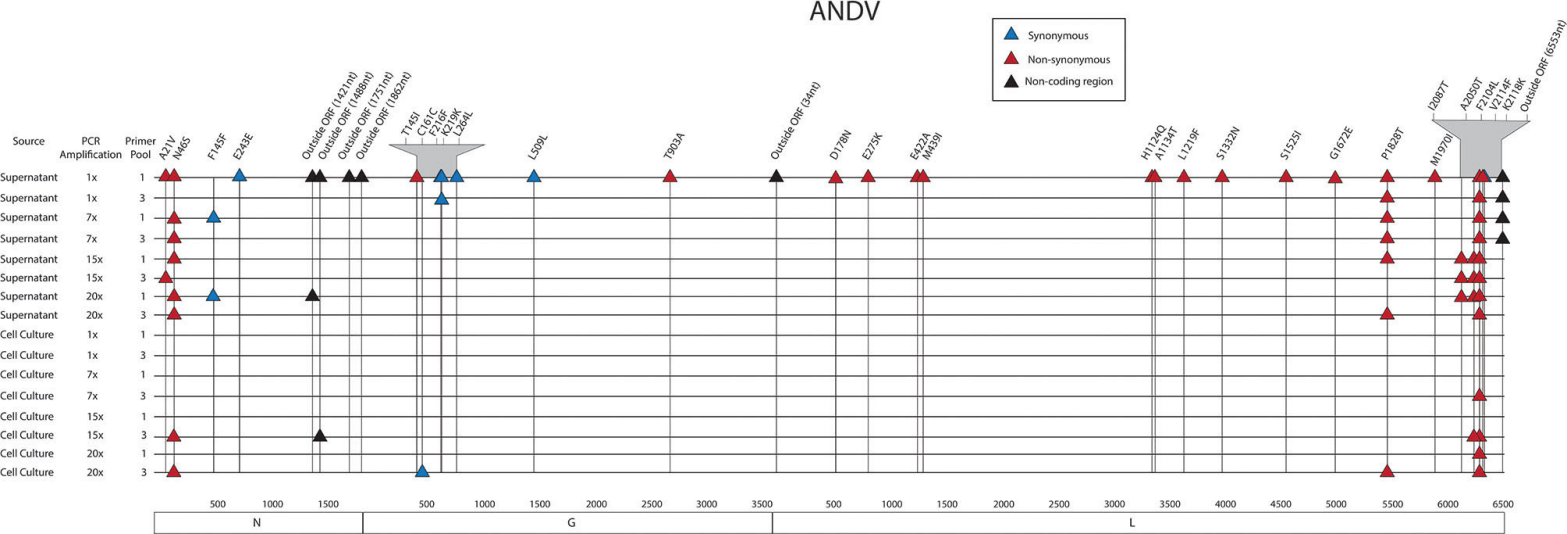
Genome mapping of SNPs of individual libraries for ANDV. Illustration shows the 16 ANDV libraries sequenced on the MiSeq and the SNPs observed across the S, M, and L genomes for each library. SNPs are distinguished by color synonymous mutations are blue triangles, non-synonymous mutations are red triangles, and SNPs found within the non-coding region are black triangles.

PCR artifacts are errors introduced during PCR amplification steps. To measure these artifacts in our pipeline, we examined ANDV sample sequences across PCR cycles and we estimated the mutation frequency of each sample based on its respective PCR cycle number (Table 4). We observed little difference in number of SNPs when using three primer pools. In fact, using multiple primer pools produces uniform mutation frequencies regardless of the number of PCR cycles. In using the multiplex primer pooling approach, we identified one instance where increased SNPs were present in the lowest PCR cycle in the sample of ANDV isolated from supernatant. There were 30 SNPs found in this library, seven in the S segment, six in the M segment, and 17 in the L segment (Figure 3). This is in contrast to previous work suggesting that lower PCR cycles reduces errors introduced by increased PCR cycles (Liu et al., 2014; Waugh et al., 2015). Our work suggests that increased PCR cycle number reduces errors amplified by a minor portion of the cDNA caused by reverse transcription. Furthermore, our approach ensures reduced introduction of PCR artifacts provided that during a multiplex primer pooling approach at least 7x PCR cycles are used.

**Table 4:** Mutation frequency of ANDV genomes amplified from supernatant or ANDV-infected Vero E6 cells and analyzed using the MiSeq.

**Source**	**Primer pool**	**(No. SNPs) mutation frequency**			
		**PCR cycle**			
		**1x**	**7x**	**15x**	**20x**
Supernatant	1	(30) 1.8 × 10^−5^	(6) 3.6 × 10^−6^	(5) 3 × 10^−6^	(6) 3.6 × 10^−6^
	3	(3) 1.8 × 10^−6^	(4) 2.4 × 10^−6^	(4) 2.4 × 10^−6^	(3) 1.8 × 10^−6^
Cells	1	N/A	N/A	N/A	(1) 5.9 × 10^−6^
	3	N/A	(1) 5.9 × 10^−7^	(4) 2.4 × 10^−6^	(4) 2.4 × 10^−6^

*Mutation frequencies were measured based on the input PFU per sample, accounting for 10% sample loss during library preparation. N/A: Not applicable; represents mutation frequency which was not estimated as parameters identifying low frequency SNPs excluded those identified in these samples*.

**Figure 4:**
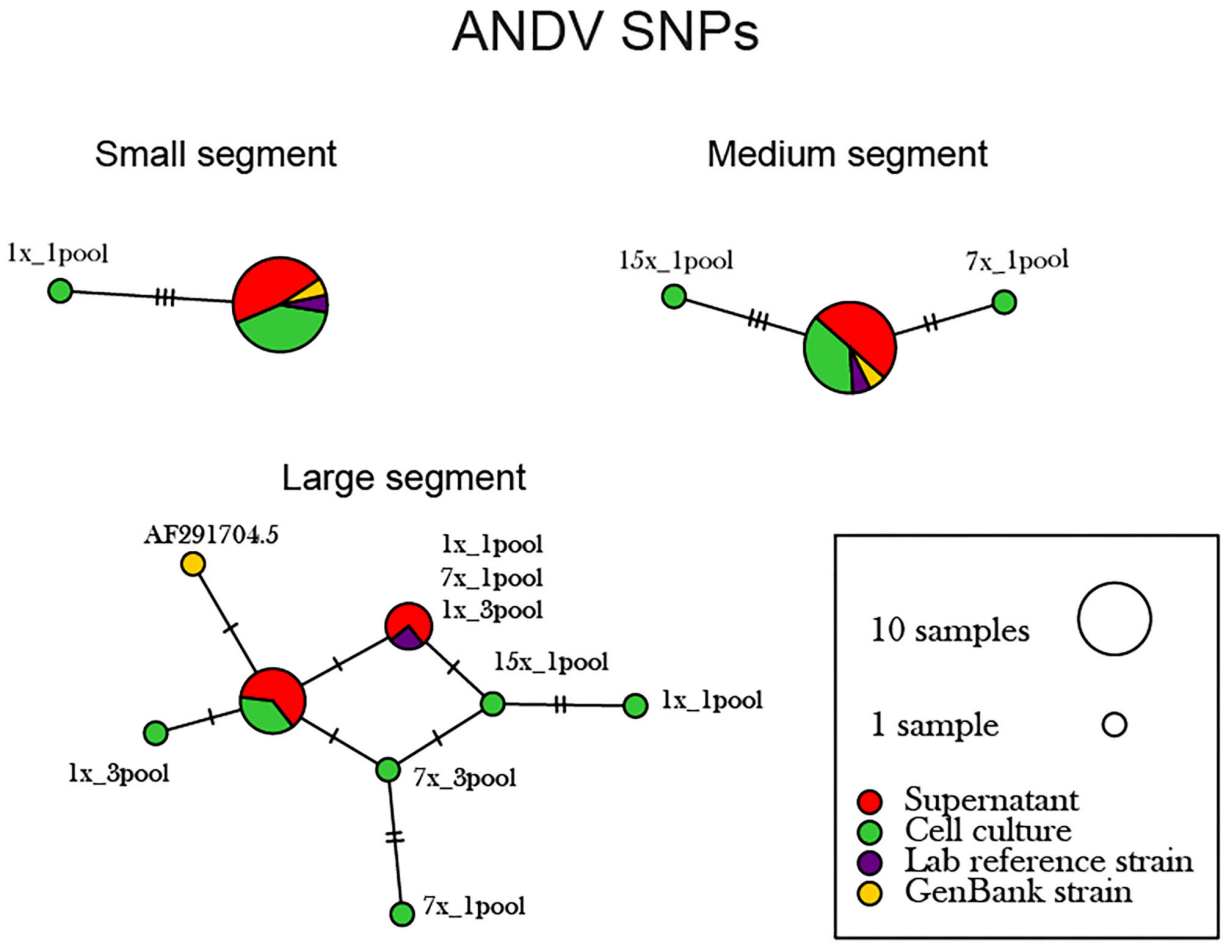
Network analysis plot of ANDV SNP differences between consensus sequences. Differences in SNP subpopulations were classified as those found within ANDV segments. Names of SNP subpopulations are defined as the number of PCR cycles and the number of primer pools involved. Colors indicated the source of each isolated virus sample, whether it originated from the cell monolayer or supernatant. Tick marks represent the number of nucleotide substitutions which differed between consensus sequences. Reference used was taken from GenBank accession no. AF291702.1, AF291703.2, AF291704.5 representing the S, M, and L genomes, respectively.

The libraries made from RNA isolated from the supernatant vs. cell-derived ANDV consensus sequences were analyzed using a network analysis approach and percent identity matrix (PIM) (Figure 4, Supplementary Table 4). Our network analysis plot and PIM constructed from ANDV consensus sequences identified that viruses sequenced from supernatant were more identical than those sequenced from cell culture. Consensus sequences derived from supernatant derived RNA showed 100% amino acid similarity compared to consensus sequences derived from cell culture which showed 97–100% similarity between consensus sequences from both reference and other preparation methods. Specifically, in the S, M, and L segments, variants emerged only from the cell culture derived amplicons. This probably reflects that amplicons from virus-infected cells are more likely to defective genomes that are not packaged.

### Two-Step Amplification of Full-Length Sequences of S, M, and L Segment vRNA of PHV Using MinION From PHV-Infected Vero E6 Supernatant or PHV-Infected Vero E6 Cells

**Figure 5:**
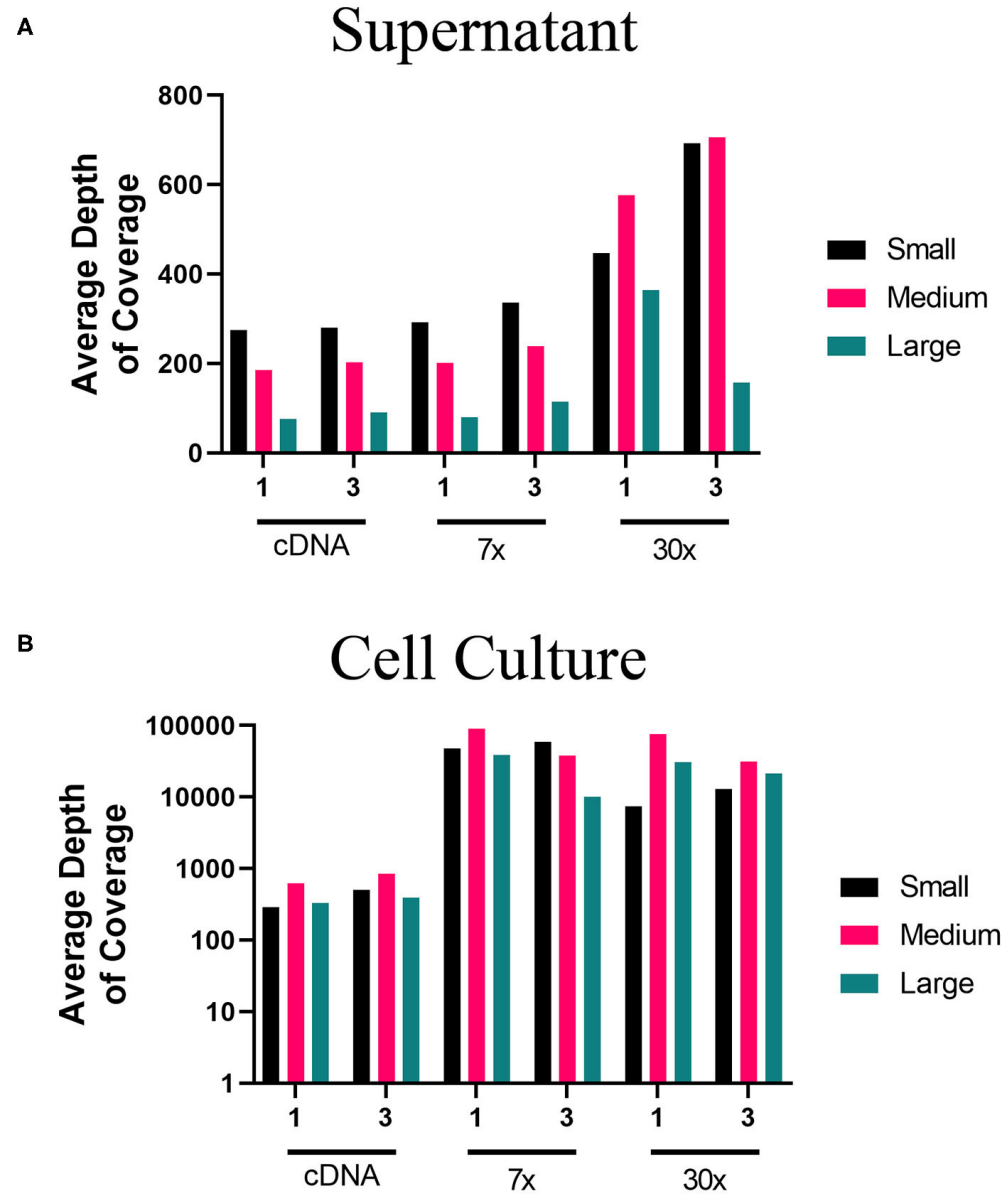
Average depth of coverage obtained from two-step MinION sequencing of PHV. Illustration shows **(A)** average depth of coverage obtained for each segment (S, M, L) of PHV RNA isolated from cell culture supernatant using different amplification approaches (cDNA, 7x, or 30x PCR cycles). **(B)** Average depth of coverage obtained for each segment of PHV RNA isolated from infected cells using different amplification approaches (cDNA, 7x, or 30x PCR cycles).

Using the MinION, we evaluated the two-step amplification of full-length sequences of S, M, and L segment vRNA using the one-pool, whole genome multiplex (Figure 1B) and the three-pool, segment-specific tiling (Figure 1C) schemes. For these experiments, we used total RNA isolated from the supernatant or cells of PHV-infected Vero E6. For each RNA source, we sequenced cDNA, 7x or 30x PCR amplification cycles. Based on the average depth of coverage, reads aligned to a greater extent from higher amplification cycles, (30x), of viral RNA amplified from supernatant (Figure 5A). Libraries made from cell culture supernatant had 97–100% genome coverage for the S segment and M segments (Supplementary Table 5). Decreased depth and coverage of the L segment was observed for libraries made from supernatant of PHV-infected cells although genome coverage was still found at >94% from directly sequencing cDNA (Figure 5A). Complete genome coverage and good depth of coverage was observed for cell-derived libraries (Figure 5B, Supplementary Table 5). Complete coverage of the S was observed regardless of the source of RNA or amplification approach (Supplementary Table 5). The M segment showed complete coverage except for those libraries prepared from supernatant where cDNA was synthesized using the one-primer pool, whole genome multiplex approach (Figure 1B). In summary, PHV libraries amplified from infected Vero E6 cells had higher genome coverage compared to cell culture supernatant (Supplementary Table 5).

The intrinsic mutation frequencies for libraries prepared from the three-pool, segment-specific tiling (Figure 1C) were similar (Table 5). In contrast, the intrinsic mutation frequencies for libraries prepared from the one-pool, whole genome multiplex (Figure 1B) had a log difference in mutation frequency between cDNA isolated from cell culture and using either 7x or 30x PCR cycles (Table 5). Except for the cDNA amplified library from cells using the one-pool method, all approaches showed a similar mutation frequency suggesting that amplification cycles did not contribute in our hands to increased error.

**Table 5:** Mutation frequency of PHV genomes amplified from supernatant or PHV-infected Vero E6 cells and analyzed using the MinION.

**Source**	**Primer pool**	**(No. SNPs) mutation frequency**		
		**PCR cycle**		
		**cDNA**	**7x**	**30x**
Supernatant	1	(9) 5.6 × 10^−5^	(9) 5.6 × 10^−5^	(13) 8.1 × 10^−5^
	3	(8) 5 × 10^−5^	(8) 5 × 10^−5^	(9) 5.6 × 10^−5^
Cells	1	(16) 1 × 10^−4^	(7) 4.4 × 10^−5^	(7) 4.4 × 10^−5^
	3	(10) 6.3 × 10^−5^	(10) 6.3 × 10^−5^	(8) 5 × 10^−5^

*Mutation frequencies were measured based on the input PFU per sample, accounting for 10% sample loss during library preparation*.

**Figure 6:**
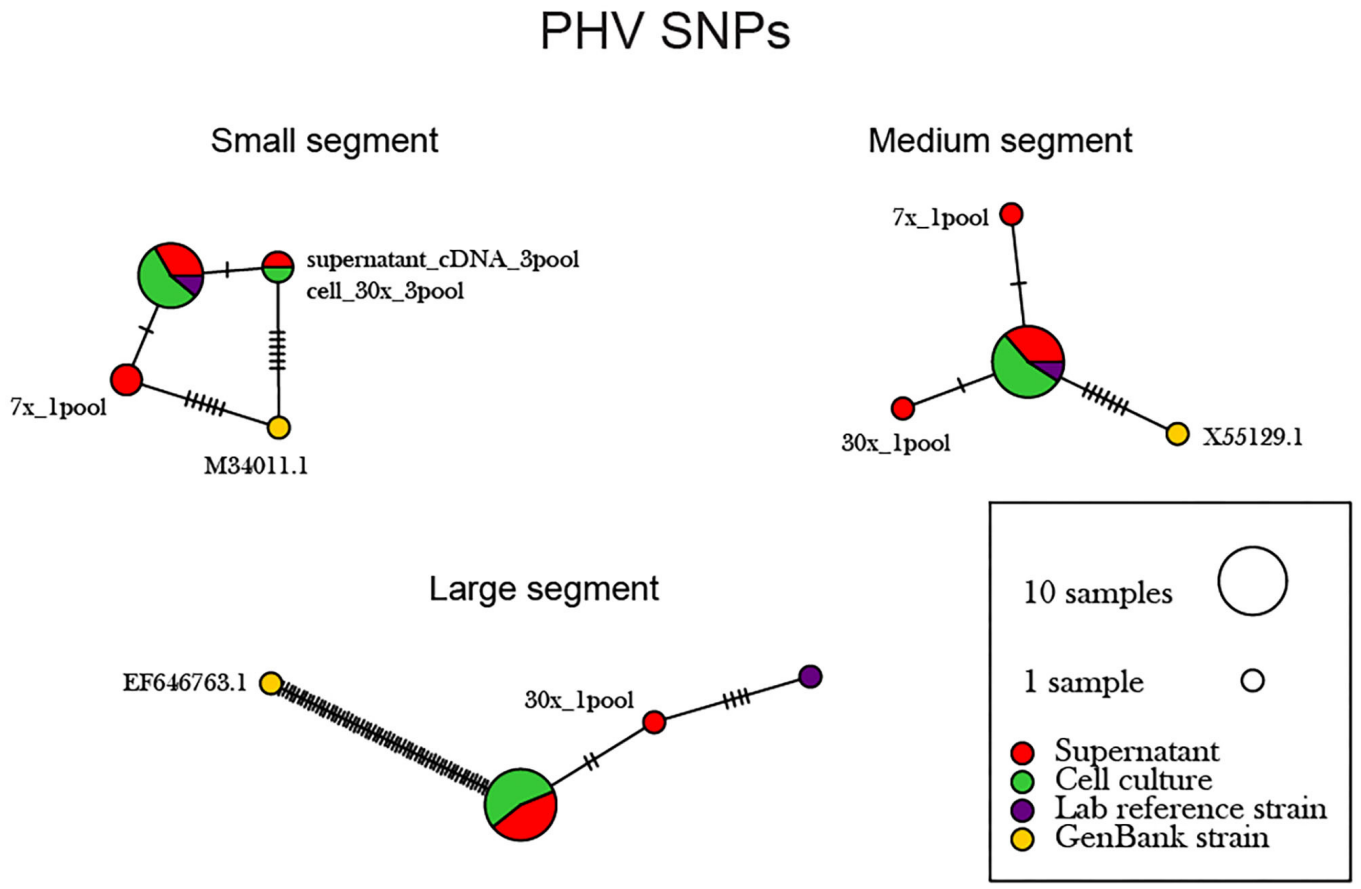
Network analysis plot of PHV SNP differences between consensus sequences. Differences in SNP subpopulations were classified as those found within PHV segments. Names of SNP subpopulations are defined as the number of PCR cycles and the number of primer pools involved. Colors indicated the source of each isolated virus sample, whether it originated from the cell monolayer or supernatant. Tick marks represent the number of nucleotide substitutions which differed between consensus sequences. Reference used was taken from GenBank accession no. M34011.1, X55129.1, EF646763 representing the S, M, and L genomes.

The supernatant vs. cell-derived PHV consensus sequences were analyzed using a network analysis approach and PIM (Figure 6, Supplementary Table 6). Our network analysis plot and PIM constructed from PHV consensus sequences identified that viruses sequenced from cells were more identical than those sequenced from supernatant. Our PIM comparison of amino acid and nucleotide variation was negligible as consensus sequences derived from supernatant showed 99.8–100% amino acid similarity compared to consensus sequences derived from cell culture which showed a 99.9–100% similarity between consensus sequences from both reference and other preparation methods. Specifically, in the S, M, and L segments, variants emerged only from the cell culture derived amplicons. However, variants were minimal across the 12 PHV consensus sequences. The L segment contained a substantial number of SNPs between the reference GenBank sequence (accession no. EF646763) and our sequenced lab strain.

### Assessment of Standing Genetic Variation of Hantaviruses With Various WGS Approaches and Evaluation Using a Network Analyses

Assessment of the standing genetic variation (SGV) inherent within a hantavirus species population will give insight into the inherent genetic plasticity within the genome. This information also provides a critical baseline for adaptation experiments *in vitro* or *in vivo*. To assess the SGV of ANDV and PHV populations in our seed stocks, we examined SNPs from two-step RT-PCR MiSeq and MinION experiments (Section two-step amplification of full-length sequences of S, M, and L segment vRNA of ANDV using MiSeq from ANDV-infected Vero E6 supernatant or ANDV-infected Vero E6 cells). SNPs that occurred in multiple libraries have a greater probability of representing the SGV within the ANDV genome. While modern MMLV-based reverse transcriptase minimize misincorporation of erroneous nucleotides, they may still become incorporated into the population-level analyses. To reduce the likelihood of calling SNPs generated by inherent error of the polymerases used in the amplification process, we used specific thresholds and cut-off values for reads. To call SNPs sequenced on the MiSeq platform we used a 1% SNP cut-off and a minimum depth of coverage of 400. To eliminate SNPs introduced through basecalling errors, we set the MinION SNP cut-off threshold at 10% and used a coverage cut-off depth of 50. In calling ANDV SNPs from the MiSeq we determined that amino acid changes which prematurely introduced a stop codon occurred at or <2% frequency (data not shown). This indicated that MiSeq SNP frequency cut-offs would better be represented at 2% using a cut-off depth of 400. Premature stop codons were not introduced using a 10% SNP frequency cut-off for the MinION.

#### Assessment of Standing Genetic Variation of ANDV Two-Step Sequenced on the MiSeq

**Figure 7:**
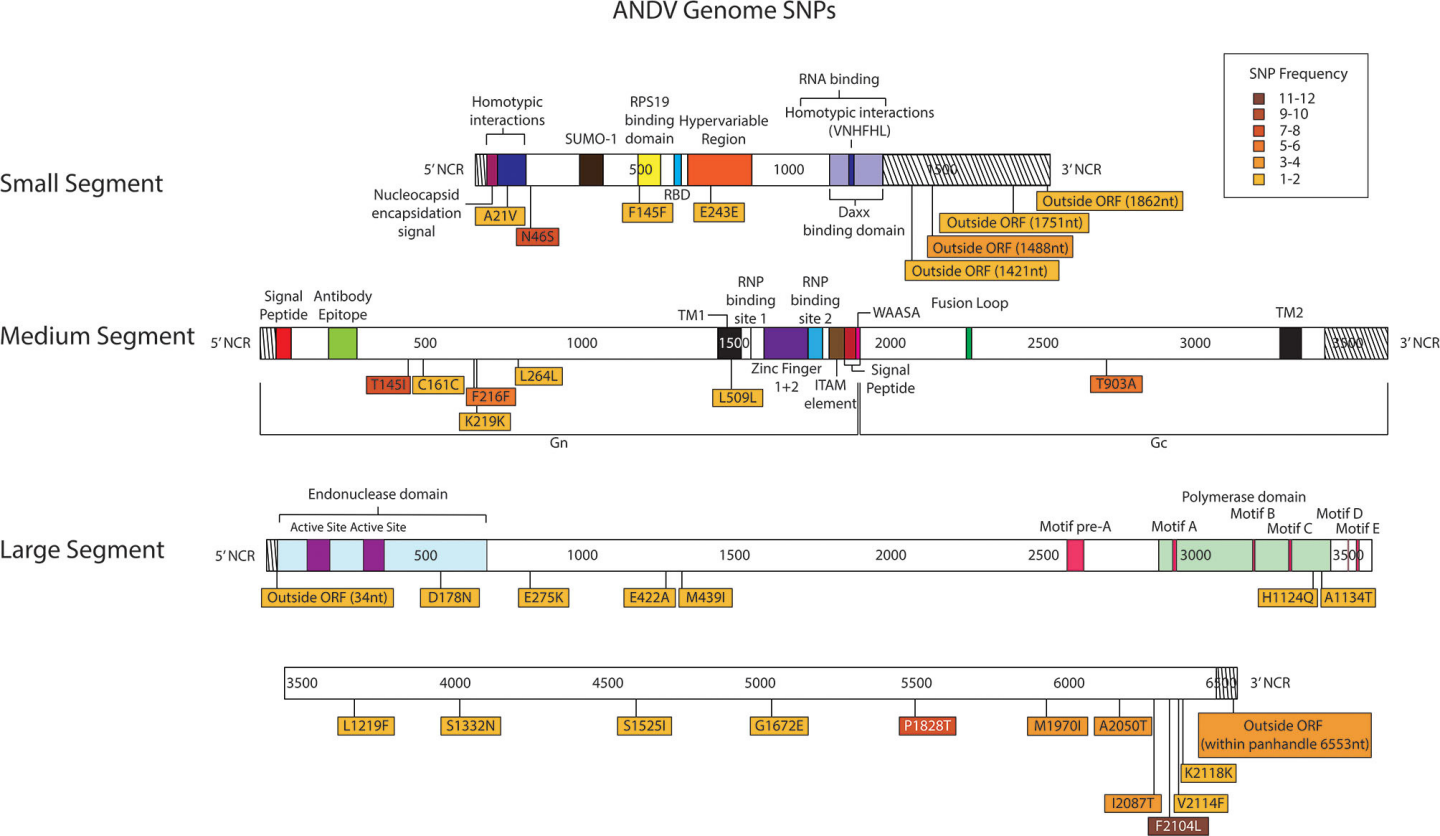
Genome mapping illustration of SNPs located within ANDV segments. Genomes are marked using nucleotide labels within each genome segment. 3' and 5' noncoding regions (NCRs) are identified at either end of each genome segment within striped boxes. SNPs are annotated by amino acid substitution and each SNP is color coded based on the number of times it was identified in any sample within the sequencing run, 16 samples were sequenced in the ANDV sequencing run. Functional domains described in the literature are annotated per each genome segment (Gott et al., 1993; Jenison et al., 1994; Plyusnin et al., 1994, 1996; Van Epps et al., 1999; Jonsson and Schmaljohn, 2001; Lober et al., 2001; Severson et al., 2001, 2005; Li et al., 2002, 2016; Shi and Elliott, 2002; Geimonen et al., 2003; Kaukinen et al., 2003; Lee et al., 2003; Tischler et al., 2003; Estrada et al., 2011; Cifuentes-Munoz et al., 2014; Ganaie et al., 2014; Rothenberger et al., 2016).

Thirty-four SNPs were identified in the 16 ANDV NGS data from MiSeq (Figure 7, Supplementary Tables 7, 8). In the ANDV genome, we identified eight SNPs within the S segment, seven SNPs in the M segment, and 20 SNPS in the L segment. In the S-segment, four SNPs were located outside the open reading frame (ORF) within the 3' NCR and four within the coding region. In the M segment no SNPS were identified in the NCR. In the L segment, one SNP was in the NCR while the other 19 were in the ORF.

In the S segment ORF, two SNPS resulted in amino acid changes, A21V, which lies in the homotypic interaction domain (Kaukinen et al., 2003), and F145F, which lies in the RPS19 domain (Figure 6) (Ganaie et al., 2014). In the hypervariable region the SNP at E243E did not result in an amino acid change. In the S segment A21V was observed twice, N46S was observed eight times, F145F was observed twice, and outside the ORF nucleotides G1421A and C1488T were each identified in two out of the sixteen libraries.

In the seven M segment ORF SNPS, five SNPS did not result in an amino acid change. Moreover, none of the M segment SNPs were observed at frequencies at or >5%. None of the amino acid changes occurred within any known functional domains (Figure 7). The M segment contained one SNP K219K which occurred in two separate libraries.

In the L segment ORF SNPS, five SNPs were observed multiple times. For example, F2104L was observed 12 times at frequencies of 42–50%, P1828T was observed seven times at 43–48% frequency, I2087T was observed four times at 10–13% frequency, A2050T was observed three times at 4–5% frequency, and G6553C was observed four times at 16–23% frequency. None of these mutations mapped to domains with any known function (Figure 7). The remaining SNPs were identified in only one of the 16 NGS data sets. Of potential importance are H1124Q and A1134T which mapped to the polymerase domain C and the D178N which mapped to the endonuclease domain (Figure 7). None of the library preparation methods presented in this paper were able to fully capture the complete SGV of the 11 ANDV SNPs repeatedly observed through each of our libraries (Figure 3). Libraries sequenced from cell culture supernatant showed that the 1x PCR cycle and one pooled method incorporated the most SGV, which included seven repeated SNPs, although this also incorporated the most artifacts (Table 4). The next method which contained the highest SGV from supernatant was the 20x PCR cycle using the one pooled method, which included six repeated SNPs. Libraries sequenced from cell culture showed that the 15x and 20x PCR cycle using the three pooled method incorporated the most SGV, which was only two SNPs.

#### Assessment of Standing Genetic Variation of PHV Two-Step Sequenced on the MinION

**Figure 8:**
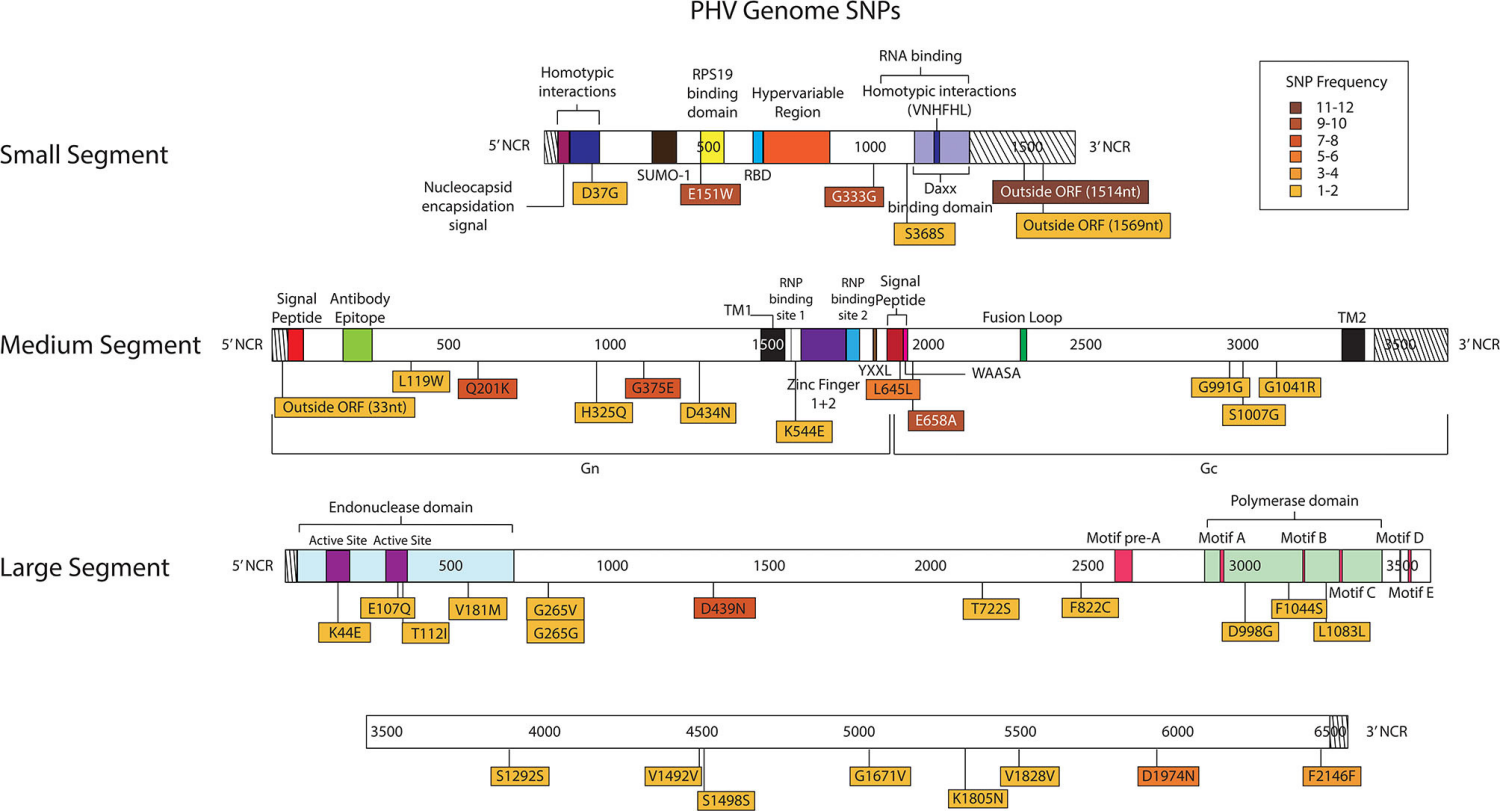
Genome mapping illustration of SNPs located within PHV segments. Genomes are marked using nucleotide labels within each genome segment. 3' and 5' noncoding regions (NCRs) are identified at either end of each genome segment within striped boxes. SNPs are annotated by amino acid substitution and each SNP is color coded based on the number of times it was identified in any sample within the sequencing run, 12 samples were sequenced in the PHV sequencing run. Functional domains described in the literature are annotated per each genome segment (Gott et al., 1993; Jenison et al., 1994; Plyusnin et al., 1994, 1996; Van Epps et al., 1999; Jonsson and Schmaljohn, 2001; Lober et al., 2001; Severson et al., 2001, 2005; Li et al., 2002, 2016; Shi and Elliott, 2002; Geimonen et al., 2003; Kaukinen et al., 2003; Lee et al., 2003; Tischler et al., 2003; Estrada et al., 2011; Cifuentes-Munoz et al., 2014; Ganaie et al., 2014; Rothenberger et al., 2016).

Thirty-eight SNPs were identified in the PHV genome (Figure 8, Supplementary Tables 9, 10). Six SNPs were in the S segment, 12 SNPs were noted in the M segment and 20 SNPs were identified in the L segment of PHV.

In the PHV S segment, two SNPs were located outside the ORF within the 3' NCR, one SNP did not have an amino acid change, and two SNPs caused amino acid changes. Three SNPs were identified multiple times, E151W was identified 10 times at frequencies of 15–38%, G333G was observed nine times at 16–76% frequency, and outside the ORF nucleotide A1514G was observed twelve times at 24–38% frequency. As in the ANDV S segment ORF, the homotypic region and the RPS19 had SNPs with amino acid changes, D37G and E151W, respectively (Figure 8).

In the M segment, one SNP was observed outside the ORF within the 5' NCR, two SNPs did not cause an amino acid change and nine SNPs resulted in an amino acid change. Four SNPs were found multiple times, Q201K was identified eight times at frequencies of 12–14%, G375E was observed seven times at 27–41% frequency, L645L was observed six times at 15–19% frequency, and E658A was observed nine times at 15–24% frequency. None of the SNPs with amino acid changes were in known functional domains (Figure 8).

In the L segment, one SNPs was observed outside the ORF, seven did not cause amino acid changes, and 13 caused amino acid changes in the ORF. Eleven SNPs were observed multiple times, E107Q was observed two times at frequencies of 17 and 21%, G265G was observed two times at a frequency of 11 and 13%, G265V was observed two times at 12 and 13% frequency, D439N was observed eight times at 24–40% frequency, T722S was observed two times at a frequencies of 11%, F822C was observed two times at a frequency of 11 and 20%, S1292S was observed two times at a frequency of 11 and 12%, V1492V was observed twice times at 39 and 48% frequency, V1828V was observed two times at a frequencies of 12%, D1974N was observed five times at 10–21% frequency, and F2146F was observed four times at 11–14% frequency. Notably, seven SNPs with amino acid changes mapped to the endonuclease domain and the polymerase domain (Figure 8).

**Figure 9:**
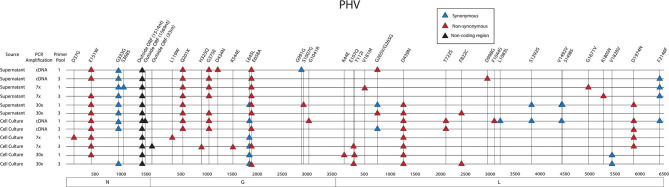
Genome mapping of SNPs of individual libraries for PHV. Illustration shows the 12 PHV libraries sequenced on the MiSeq and the SNPs observed across the S, M, and L genomes for each library. SNPs are distinguished by color synonymous mutations are blue triangles, non-synonymous mutations are red triangles, and SNPs found within the non-coding region are black triangles.

As previously shown with ANDV, none of the library preparation methods used with PHV were able to fully capture the complete SGV of the 18 SNPs repeatedly observed through each of our libraries (Figure 9). Libraries sequenced from cell culture supernatant showed that the 30x PCR cycle using the one pooled method incorporated the most SGV, which included 11 repeated SNPs. Libraries sequenced from cell culture showed that the cDNA sequenced with either the one or three pooled method incorporated the most SGV, which was 10 SNPs, although the one pooled method also incorporated more artifacts (Table 5). Secondary to this, the 30x PCR cycle using the three pooled method incorporated the next highest number of SNPs contributing to the SGV of PHV which was eight SNPs.

## Discussion

Herein, we have designed, tested and compared several NGS methods for detection and genetic evaluation of full-length genome sequences of hantaviruses. These versatile NGS pipelines may be employed to address various questions in clinical diagnostic research and research focused on the structure of viral populations and evolution. While we focused on the hantaviral genome, these approaches can be readily translated to other single-stranded, segmented RNA viruses. Moreover, we show that these methods can be used to identify low frequency SNPs which are critical for understanding the effect of SNPs on viral phenotypes. Critically, these methods should allow the robust evaluation of the standing genetic variation of viruses in reservoir hosts or spillover infections. The accurate identification of SNPs in viral populations is essential to building an accurate profile of virus in nature and predicting those variants which may pose a risk to public health in outbreaks of emerging viruses.

Primer sets having a large panel of short amplicons have been developed for use in sequencing viral genomes obtained from tissue or patient specimens. This method, termed RNA jackhammering, was made popular by Worobey et al. and has greatly aided the sequencing of samples with degraded viral RNA. Originally this method was designed for archived specimens and has been used to a large extent for tissue specimens in the last few years (Worobey et al., 2016). This approach is similar to the amplicon-based approach reported by No et al. (2019) in which they successfully identified HTNV genomes from *Apodemus agrarius* tissue using 118 primer sets to generate short 150 bp amplicons that covered the genome. In this approach, viral RNA present at a higher Ct value in the tissue had reduced sequencing background, but as viral titer decreased, non-viral reads increased; yet complete genome coverage was still attained at 10^2^ copies. We report a simpler tiling approach using only 23–24 primer sets of overlapping 500–1 kb amplicons to cover each viral genome. Similar to RNA jackhammering approaches, we show that longer amplicons provided excellent coverage and depth, but the pooling and multiplexing of the primers was critical.

In assessing other library preparation methods, Kugelman et al., compared target-capture, SISPA and amplicon-based approaches for whole genome sequencing of Ebola virus (EBOV) virus and reported that target capture is the optimal methodology for sensitivity and sample preparation error (Kugelman et al., 2017). They showed SISPA methods contained an error rate of 4.4 × 10^−5^ error/site/copy for EBOV, while target capture enrichment methods show 1.4 × 10^−5^ error/site/copy. In contrast No et al. showed that for HTNV, SISPA is less efficient in regards to genome coverage with 10^5^ genome copies having <50% genome coverage of L and M segments and <75% genome coverage of S segment (No et al., 2019). In agreement with Kugelman et al., No et al. concluded that target capture methods provided complete genome coverage of HTNV with as low as 10^3^ copies. In our one-step approach using one forward and one reverse primer for each segment, we obtained full-length genome coverage with libraries made from 10^3^ virions of ANDV, DOBV, or HTNV, and we showed 99–100% genome coverage across the S, M, and L segments. In sequencing of libraries for ANDV on the MiSeq we observed an intrinsic mutation frequencies of 10^−6^ for most samples (Table 4). MinION sequencing of PHV samples showed an intrinsic 10^−5^ level of mutation frequencies (Table 5). This is similar to the intrinsic mutation frequency reported by our laboratory and others as well as mirroring mutation frequencies previously determined for other RNA virus genomes (Holland et al., 1992; Severson et al., 2003; Chung et al., 2013). The highest mutation frequencies observed from ANDV and PHV, 1.8 × 10^−5^ and 1 × 10^−4^, respectively, were observed with the 1x PCR cycle or cDNA samples, respectively. This suggests that lower PCR cycles lead to higher mutation frequencies between samples. Previous research has supported the idea of high genomic integrity maintained within *Oligoryzomys*-borne viruses such as ANDV (Padula et al., 2000). Although these viruses have been propagated and have adapted to Vero E6 cells, further study is required to define the SGV in natural isolates. For example, SNPs which caused amino acid changes in cases of ANDV may differ from the SGV of ANDV isolated from the natural reservoir (Tischler et al., 2003).

It is interesting to note that at the 3' and 5' ends of each genome 20–22 nucleotides make up the putative panhandle regions. We identified four SNPs within the 3' non-coding region (NCR) of the S, one in the L and one in the 5' NCR of the L were identified in ANDV (Figure 7). In addition, we identified two S segment SNPs in the 3' NCR and one M segment SNP in the 5' NCR of PHV (Figure 8). Secondary RNA structure within the untranslated region has been shown to effect translation in Dengue virus and transcription in Influenza A virus (Manzano et al., 2011; Liu et al., 2015). Furthermore, a change in virulence was observed in newborn mice infected with HTNV having an amino acid change in the M segment and a nucleotide change in the 3' NCR of the L segment (Ebihara et al., 2000).

In this article we have identified a variety of library preparation amplification approaches from supernatant or cell culture. In fact, greater population diversity evidenced by the SGV of low-frequency SNPs are found in higher PCR cycles (20x or 30x) in the supernatant (Figures 3, 9), whereas low frequency mutations are often lost in cell culture (Supplementary Tables 7–10). This suggests that virus particles released outside the cell in the supernatant show greater SGV than what is observed inside of the cell and this is most noticeable in higher PCR cycles. Virus within the supernatant represent infectious virus-like particles (VLPs) whereas VLPs found in cell culture are more likely to incorporate false artifacts within their genomes through the RdRp gene.

One limitation we identified with the whole segment PCR (Figure 1A) using one-step RT-PCR, is that the L segment required amplified in two individual PCR reactions (~3.6 kb max.). Another limitation is that this one-step reaction does not distinguish between mRNA, cRNA, or vRNA in specimens. In contrast to the two step, the one-step RT-PCR of PHV and SNV using the MinION gave a greater number of aligned reads, average depth of genome and genome coverage for the S, M, and L genomes of SNV as well as the S and M genomes of PHV. Hence if sensitivity is required the one step RT-PCR may be the best choice. However, in selection of the MinION, the high error rate due to homopolymer region must also be considered. The current version of nanopore (R9.4) faces a homopolymer sequencing problem. As of January 2020, ONT has manufactured R10.3 for public availability, a new version of nanopore, which has a longer barrel and a dual reader head, enabling improved resolution of homopolymeric regions and improving the consensus accuracy of sequencing data. Applying the new version pore to sequence amplicon sample would resolve this issue. A second consideration is that the sequencing chemistry of Illumina includes amplification steps; thus, the throughput will be much higher with Illumina than with nanopore sequencing, which does not amplify the DNA molecules in the library preparation step. The newest nanopore version (R10.3) has been claimed to generate higher throughout and should be tested for improvement in sequencing.

Although ONT platform allows vRNA detection within minutes of initiation of sequencing, this technology remains in early phases in application to clinical specimens. One benefit observed of the long-read data output from the MinION was that 92% genome coverage was achieved from only 23 reads aligned to the M segment of PHV (Table 2). Longer reads produced by ONT platforms may be more beneficial for more complete genome coverage from viral samples isolated from tissue, although shorter reads observed with the Illumina platform would provide greater depth of coverage across the genome. In comparing PHV sequencing from virus-infected cell culture supernatant or virus-infected cells, the number of reads aligning to the genome as well as the average depth of coverage was greater from RNA isolated from virus-infected cell sample than for supernatant (virus seed stock) (Supplementary Table 5).

A final consideration in the choice of the pipeline used is often the length of time. In comparing one-step and two-step approaches, the one-step approach took <1 h to run on the gradient cycler, while the two-step approach took 20 min for cDNA synthesis and between 8 min to run the 1x PCR cycle to 50 min to run the 30x PCR cycle. The entire MinION pipeline to sequence 12 samples from the start of library preparation to loading the library on the flow cell took ~8 h. In comparison, the length of time to prepare 16 samples to be run on the MiSeq took ~12 h from the start of library preparation to loading the final library into the cartridge.

In conclusion, the methods reported herein provide a WGS roadmap for the robust and accurate identification and sequencing of hantaviruses. Importantly, they provide an approach to accurately measure the SGV of hantaviruses in nature. Our future efforts will employ these standardized methods to the epidemiology of hantaviruses in rodent reservoirs to gain insight into their diversity in nature.

## Data Availability Statement

The datasets generated for this study can be found in the NCBI SRA repository https://www.ncbi.nlm.nih.gov/bioproject/PRJNA628955/.

## Author Contributions

CJ, IN, EW, and MT contributed conception and design of the study. MT, TW, and EW performed wet lab benchwork. PJ, EW, and MT contributed to computational and bioinformatic analyses. All authors contributed to manuscript revision, read, and approved the submitted version.

## Conflict of Interest

The authors declare that the research was conducted in the absence of any commercial or financial relationships that could be construed as a potential conflict of interest.
