# Corrigendum: Amplicon-Based, Next-Generation Sequencing Approaches to Characterize Single Nucleotide Polymorphisms of Orthohantavirus Species

**DOI:** 10.3389/fcimb.2021.804914

**Published:** 2022-01-24

**Authors:** Mariah K. Taylor, Evan P. Williams, Thidathip Wongsurawat, Piroon Jenjaroenpun, Intawat Nookaew, Colleen B. Jonsson

**Affiliations:** ^1^ Department of Microbiology, Immunology and Biochemistry, The University of Tennessee Health Science Center, Memphis, TN, United States; ^2^ Department of Biomedical Informatics, College of Medicine, University of Arkansas for Medical Sciences, Little Rock, AR, United States

**Keywords:** hantavirus, Orthohantavirus, NGS, next generation sequencing, MinION, illumina MiSeq, single nucleotide polymorphism, SNP

## Error in Figure/Table

In the original article, there was a mistake in **Table 3** as published. **The data under columns for the header “Large segment” and the header “Small segment” were mistakenly switched prior to submission**. The corrected **Table 3** appears below.

**Table 3:** Percent genome coverage of ANDV Illumina MiSeq run using a 400x minimum coverage depth.

Sample Source	PCR Cycle	Pool	Large Segment	Medium Segment	Small Segment	
			% Genome Coverage		
Supernatant	1x	1	100%	96%	98%
		3	100%	97%	96%
	7x	1	100%	96%	99%
		3	0%	98%	99%
	15x	1	0%	83%	99%
		3	0%	82%	99%
	20x	1	0%	82%	99%
		3	0%	96%	98%
Cells	1x	1	0%	0%	0%
		3	0%	0%	0%
	7x	1	0%	0%	0%
		3	0%	0%	0%
	15x	1	0%	0%	0%
		3	0%	82%	97%
	20x	1	0%	0%	0%
		3	0%	82%	96%

For those samples stated as “3 pool”, the sequencing strategy ( **Figure 1D**) used overlapping amplicons for each segment, however primers were placed into one of three disjointed primer pools. The multiplexed amplicons were purified and normalized before the 3 pools were mixed for preparation of Nextera XT libraries. In contrast, for those samples stated as “1 pool”, all amplicons were synthesized in one reaction in **Figure 1B** (one pool) and purified for preparation of Nextera XT libraries.

The authors apologize for this error and state that this does not change the scientific conclusions of the article in any way. The original article has been updated.

In the original article, there was an error. **This error included a statement which described the incorrect Table 3 in the Results section**.

A correction has been made to ***Results***, ***Two-Step Amplification of Full-Length Sequences of S, M, and L Segment vRNA of ANDV Using MiSeq From ANDV-Infected Vero E6 Supernatant or ANDV-Infected Vero E6 Cells, Paragraph number 5***:

**We used a 400x depth of coverage threshold to assess ANDV genome coverage** (**Table 3**). **The supernatant samples which gave the highest genome coverage across all segments was the 1x PCR and 7x PCR. The samples from infected cells showed good coverage at 15x and 20x PCR cycles although this was only true for the S and M segments as the L segment provided no coverage at this depth**.

The authors apologize for this error and state that this does not change the scientific conclusions of the article in any way. The original article has been updated.

## Publisher’s Note

All claims expressed in this article are solely those of the authors and do not necessarily represent those of their affiliated organizations, or those of the publisher, the editors and the reviewers. Any product that may be evaluated in this article, or claim that may be made by its manufacturer, is not guaranteed or endorsed by the publisher.

